# Prevalence of episiotomy and associated factors among women who gave birth at public health facilities in Jigjiga town, eastern Ethiopia: A cross-sectional study

**DOI:** 10.1371/journal.pgph.0003216

**Published:** 2024-05-20

**Authors:** Tamene Belay, Agumasie Semahegn, Haymanot Mezmur, Teshale Mulatu

**Affiliations:** 1 Department of Midwifery, College of Medicine and Health Sciences, Jigjiga University, Jigjiga, Ethiopia; 2 School of Nursing and Midwifery, College of Health and Medical Sciences, Haramaya University, Harar, Ethiopia; 3 Centre for Innovative Drug Development and Therapeutic Trials for Africa (CDT-Africa), College of Health Sciences, Addis Ababa University, Addis Ababa, Ethiopia; 4 Department of Population, Family and Reproductive Health, School of Public Health, University of Ghana, Accra, Ghana; PLOS: Public Library of Science, UNITED STATES

## Abstract

**Introduction:**

Maternal morbidity and mortality has remained a major public health concern worldwide. Basic emergency obstetric care is the primary intervention to prevent obstetric complications and maternal death. Episiotomy is one of the basic obstetrical procedures used to facilitate vaginal delivery, shorten the second stage of labor and prevent complications. However, there is a paucity of evidence on the prevalence and factors associated with episiotomy among women who gave birth in eastern Ethiopia.

**Objective:**

This study aimed to determine the prevalence of episiotomy and its associated factors among women who gave birth at public health facilities in Jigjiga town, eastern Ethiopia.

**Methods:**

A facility-based cross-sectional study was conducted among women who gave birth vaginally from May 1 to June 30, 2022. A total of 422 study participants were recruited using systematic random sampling. Data were collected using structured questionnaires through a face-to-face interview supported with standard observational checklist and reviewing medical records. A logistic regression analysis was carried out to examine the association between explanatory variables and episiotomy. An adjusted odds ratio (AOR) at a 95% confidence interval (CI) at a P-value <0.05 was used to declare significant association.

**Results:**

The prevalence of episiotomy among women was 52.6% (95% CI: 47.8%, 57.0%). Obstetric complications during current pregnancy (AOR:3.92, 95% CI: 1.59, 9.68), birth weight ≥4000 gm (AOR: 4.30, 95% CI: 1.53, 12.04), induction of labor (AOR: 3.10, 95% CI: 1.62, 5.93), meconium-stained amniotic fluid (AOR:2.10, 95% CI: 1.14, 3.88), duration of the second stage of labor ≥90 minutes (AOR:3.09, 95% CI: 1.53, 6.23), instrumental delivery (AOR: 2.69, 95%, CI: 1.39, 5.19), and female genital mutilation (AOR: 2.91, 95% CI: 1.83, 4.64) were factors significantly associated with episiotomy.

**Conclusion:**

Slightly more than half of the women who gave birth at public health facilities in the study area underwent episiotomies. In addition to the common obstetric factors, having a female genital mutilation scar increased the risk of women’s experiencing episiotomies. Therefore, intervention should be tailored to address the identified obstetric risk factors and avoid female genital mutilation in the community to reduce women’s experiences of episiotomies in the future.

## Introduction

Globally, 140 million childbirths occur annually and the majority of them are vaginal deliveries [1]. Episiotomy is a surgical incision performed to widen the soft tissue of the birth canal to facilitate vaginal delivery and shorten the second stage of labor. It is one of the most commonly performed procedures in basic emergency obstetrics care [2–4].

According to World Health Organization (WHO) estimates, Ethiopia, India, Nigeria, Pakistan, Afghanistan, and the Democratic Republic of Congo were among the six countries with high maternal deaths that contribute to 50% of maternal deaths globally [5,6]. In Ethiopia, 412 women deaths per 100,000 live births and the neonatal mortality rate is 33 per 1,000 live births [5,6].

Postpartum hemorrhage is the leading cause of maternal mortality that accounts for 32% of maternal deaths in low and middle income countries [7,8]. Genital tract trauma is one of the causes for postpartum hemorrhage that triggers maternal death in Nigeria [8], Denmark [9], and Ethiopia [10].

Episiotomy has considerable benefit in preventing genital tract trauma or perennial tears and adverse delivery outcomes [11,12]. However, episiotomy has been discouraged from routine practice because of its increased risk of complications such as perennial pain and laceration, episiotomy dehiscence, hemorrhage, anal sphincter injury, rectal mucosal damage, chronic pain, scar, anorectal dysfunction, urinary incontinence, pelvic organ prolapses, and sexual dysfunction [13–15].

The WHO recommends healthcare providers reduce episiotomy rates below 10% [16], but episiotomy practiced commonly and its prevalence ranges from 9.7% [17] to 96.2% worldwide [18]. In Ethiopia, the prevalence of episiotomy varies from 35.2% [19] to 68% [20]. Prolonged second stage of labor, low parity and younger maternal age, shoulder dystocia and occiput posterior position, labor management personnel, and Female Genital Mutilation (FGM) were some of the identified factors associated with episiotomy practice [21–24].

Though the level of episiotomy in developed countries is dropping tremendously, it is still high in developing countries like Ethiopia. Understanding the level of episiotomy and associated factors in a particular setting is crucial to guide healthcare professionals and health policymakers to adopt evidence-based restrictive episiotomy practice.

Even though there are studies on episiotomy in the eastern part of the country, the previous studies have not assessed the association of FGM with episiotomy rate in the Somali regional state where FGM is common. Furthermore, few published evidences in the study area relied on the secondary analysis of medical records only [25]. Therefore, the main aim of this study was to assess the prevalence of episiotomy and its associated factors among laboring women who gave birth vaginally at public health facilities in Jigjiga town, Somali Regional State, eastern Ethiopia using primarily collected data complemented with observational checklist and medical record review.

## Methods

### Study design and setting

A facility-based cross-sectional study was conducted in public health facilities in Jigjiga town, Somali Regional State, Eastern Ethiopia, from May 1 to June 30, 2022, which is located about 628 km east of Addis Ababa. The town contains 30 sub-districts or kebeles (the smallest structural administrative unit in Ethiopia), of which 20 are urban and 10 are rural. There were approximately 17,001 households with an estimated total population of 426,122. The town has three public Hospitals and one health center (Jigjiga University Sheik Hassen Yebere referral Teaching Hospital, Karamara General Hospital, Ablele Primary Hospital, and Ayerdega Health Centre) that provide maternal and child health care services [26].

### Population and sampling

**Fig 1:**
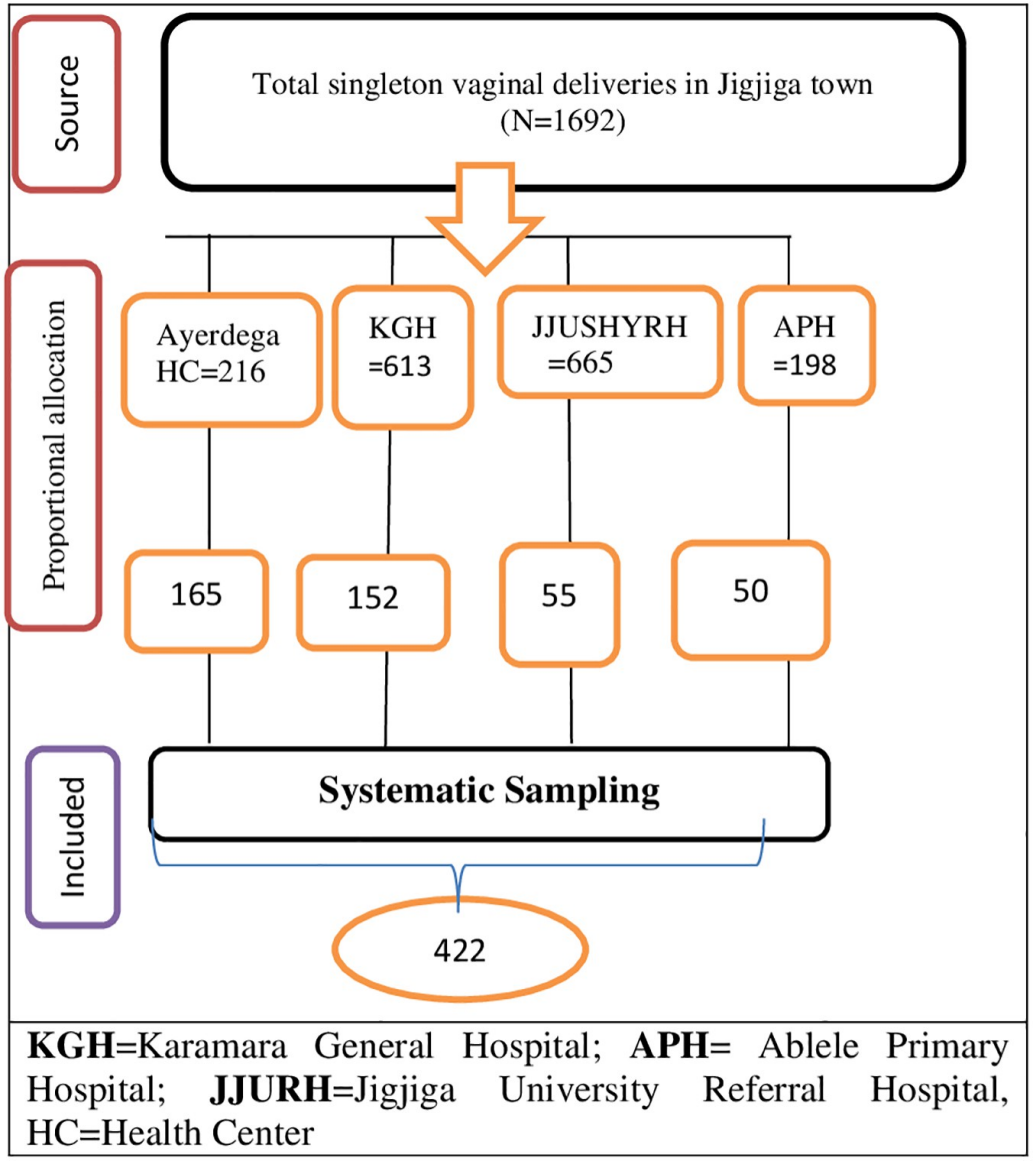
Schematic presentation of sampling procedure at public health facilities in Jigjiga town, 2022.

Women who gave birth vaginally at public health facilities in Jigjiga town during the data collection period were eligible for the study. The required sample size for this study was determined using a single population formula by considering the following parameters: 95% confidence interval, 5% margin of error, the proportion of episiotomy practice (48%) [24], and an added 10% non-response rate. The final calculated sample size was 422. A total of 1,692 women gave birth through vaginal delivery over the previous two months in the public health facilities in Jigjiga town. The sample size was allocated proportionally to each public health facility (Fig 1).

A systematic random sampling technique was used to recruit the study participants from each facility.

The interval (K^th^) for the systematic random sampling method was obtained dividing the total number of women who gave birth by the sample size which yielded four (1,692 /422 = 4). The first woman was selected by lottery method using random table (number) among those women who gave birth at respective health facilities, and study participants were identified systematically every fourth interval until the desired sample size fulfilled.

### Data collection tool and method

The questionnaire was adapted from various literatures [19,27–29]. The questionnaire was initially drafted in English, translated into local languages (Amharic and Af Somali), and then back translated to English to check for consistency. Data were collected through face-to-face interviews using a structured questionnaire and an observation checklist that contained women’s socio-demographic traits, maternal and fetal conditions, labor and delivery-related characteristics, FGM status and episiotomy experience of the women. Two days of training were given to the data collectors by the principal investigator on the objectives, how to use the checklist, fill out the questionnaire, and how to handle respondents. Data were collected by six trained midwives who have Bachelors of Science in midwifery and supervised by four postgraduate nurses in field of maternal health at the respective health facilities. The tool was pretested at another hospital which is found near to the study area. The tool’s reliability was evaluated by examining the internal consistency of the items (Cronbach’s α = 0.86) and validity was evaluated through content validity.

All data collectors observed the women from the onset of the active first stage of labor until the outcome occurred, after that, secondary data extraction is carried out till the women’s condition is stabilized. The medical records were reviewed to extract information about fetal conditions (gestational age, fetal presentation, amniotic fluid status and fetal heart rate pattern, birth weight), the onset of labor; obstetric complications in the current pregnancy; duration of the second stage of labor; mode of delivery; birth outcome, time of delivery; birth attendant’s professional status; and indications of episiotomy practice. The data collectors used a unique numeric identifier to track the mothers’ card back during data extraction. The interview was conducted post-partum after the mothers were stabilized; permissions to be to be interviewed and being observed throughout the data collection process were received from all study participants.

Maternal FGM status and episiotomy in the current delivery were ascertained by using observation checklist. The code of participants for the interview was linked with their medical records and there is no any personal identifiers used for the purpose of the study. The interview and observation took place where the mother’s privacy and comfort was kept.

### Data processing and analysis

Collected data were entered into computer using EpiData 3.1 and exported to SPSS (v26) for clean analysis. Descriptive statistical analysis results were represented by proportions, frequency, mean, and standard deviation. Binary and multivariable logistic regressions were used to examine association between the outcome variable (episiotomy) and independent (explanatory) variables. Those explanatory variables with a P-value ≤0.25 in the bi-variable analysis were included into the multivariable logistic regression analysis to control for confounding effect. The Hosmer-Lemeshow goodness of fit test was used to fit the model. The adjusted Odd Ratio (AOR) at 95% confidence interval (CI) was used to measure the strength of the association, and a P-value less than 0.05 was taken as significant in the final model.

### Ethical considerations

Ethical clearance to conduct the study was obtained from Haramaya University, College of Health and Medical Sciences, Institutional Health Research Ethics Review Committee with reference number IHREC/070/2022. The study was conducted in accordance with the Declaration of Helsinki. Informed verbal and written consent was obtained from each study participant on a voluntary basis to be included in the study. Informed consent from the participant or legal guardian and assent from them were obtained for study participants younger than 18 years old and for those who have not attended formal education. Collected data was kept confidential anonymously.

## Results

### Socio-demographic characteristics of study participants

A total of 422 women participated in this study. The mean age of the participants was 28.4 (± 4.8) years and more than half of the study participants 275 (65.2%) were in the age group of 25 to 34. Concerning the participants’ residence, the majority 317 (75.1%) were urban residents, and almost all 407(96.5%) of the women were married. More than one-third, 157 (37.2%) of the study participants were unable to read and write, and 196(46.4%) of them were housewives. More than half of 247 (58.5%) of them were Muslims (Table 1).

**Table 1:** Socio-demographics characteristics of the participants at public health facilities in Jigjiga town, Somali regional state, eastern Ethiopia, 2022 (n = 422).

Variables	Categories	n	%
**Age group(years)**	15–24	96	22.7
	25–34	275	65.2
	35–49	51	12.1
**Marital status**	Married	407	96.5
	Divorced	11	2.6
	Widowed	4	0.9
**Age at 1^st^ marriage**	<18 years	40	9.5
	≥ 18years	382	90.5
**Educational status**	Unable to read and write	157	37.2
	Can read and write but no formal education	145	34.4
	Primary	74	17.5
	Secondary and above	46	10.9
**Ethnicity**	Somali	212	50.2
	Amhara	104	24.7
	Oromo	71	16.8
	Others^**a**^	35	8.3
**Occupation**	housewife	196	46.4
	Government employee	34	8.1
	Merchant	165	39.1
	Farmer	17	4.0
	Others^**b**^	10	2.4
**Religion**	Muslim	247	58.5
	Orthodox	111	26.3
	Protestant	64	15.2
**Residence**	Urban	317	75.1
	Rural	105	24.9

*Others ^**a**^; Sidama, Gurage, Wolayta and Tigray; *Others^**b**^; Non-governmental Organization, student and daily laborer.

### Maternal and fetal related characteristics

The majority of the women were multigravida (81.8%, n = 345). Three-fourths (74.9%, n = 316) of women had ANC visits of which only 92(29.1%) of them had 4 ANC visits and above. Thirty-six (8.5%) of the women had an obstetric complication during the current pregnancy. Two-thirds (66.1%, n = 279) of the women who gave birth had FGM scars (Table 2).

**Table 2:** Maternal characteristics of women gave birth at public health facilities in Jigjiga town, Somali regional state, eastern Ethiopia, 2022 (n = 422).

Variables	Categories	N	%
**Gravidity**	Primigravida	77	18.2
	Multigravida	345	81.8
**parity**	primipara	83	19.7
	Multipara	339	80.3
**History of instrumental delivery**	Yes	114	33.6
	No	225	66.4
**Instrument type (n = 114)**	Vacuum	90	78.9
	Forceps	24	21.1
**History of episiotomy**	Yes	112	33.0
	No	227	67.0
**History of obstetric complication**	Yes	21	6.2
	No	318	93.8
**Complication type (n = 21)**	Gestational HTN	9	42.9
	Gestational DM	5	23.8
	Vaginal bleeding	7	33.3
**Birth spacing**	≤ 2 years	169	49.9
	> 2 years	170	50.1
**History of abortion**	Yes	32	9.3
	No	313	90.7
**Number of abortion (n = 32)**	<2 times	17	53.1
	≥2 times	15	46.9
**ANC follow-up**	Yes	316	74.9
	No	106	25.1
**Number of ANC visit (n = 316)**	First visits	13	4.1
	Second visits	118	37.3
	Third visits	93	29.4
	Fourth visit and above	92	29.1
**Current pregnancy complication**	Yes	36	8.5
	No	386	91.5
**Type of complication (n = 36)**	Gestational HTN	16	44.5
	Gestational DM	7	19.4
	Vaginal bleeding	10	27.8
	Iron deficiency anemia	3	8.3
**Age at 1st pregnancy**	≥20 years	371	87.9
	<20 years	51	12.1
**FGM**	Yes	279	66.1
	No	143	33.9

**Note:** ANC: Antenatal care; FGM: Female Genital mutilation; HTN: Hypertension; DM: Diabetes Mellitus.

**Fig 2:**
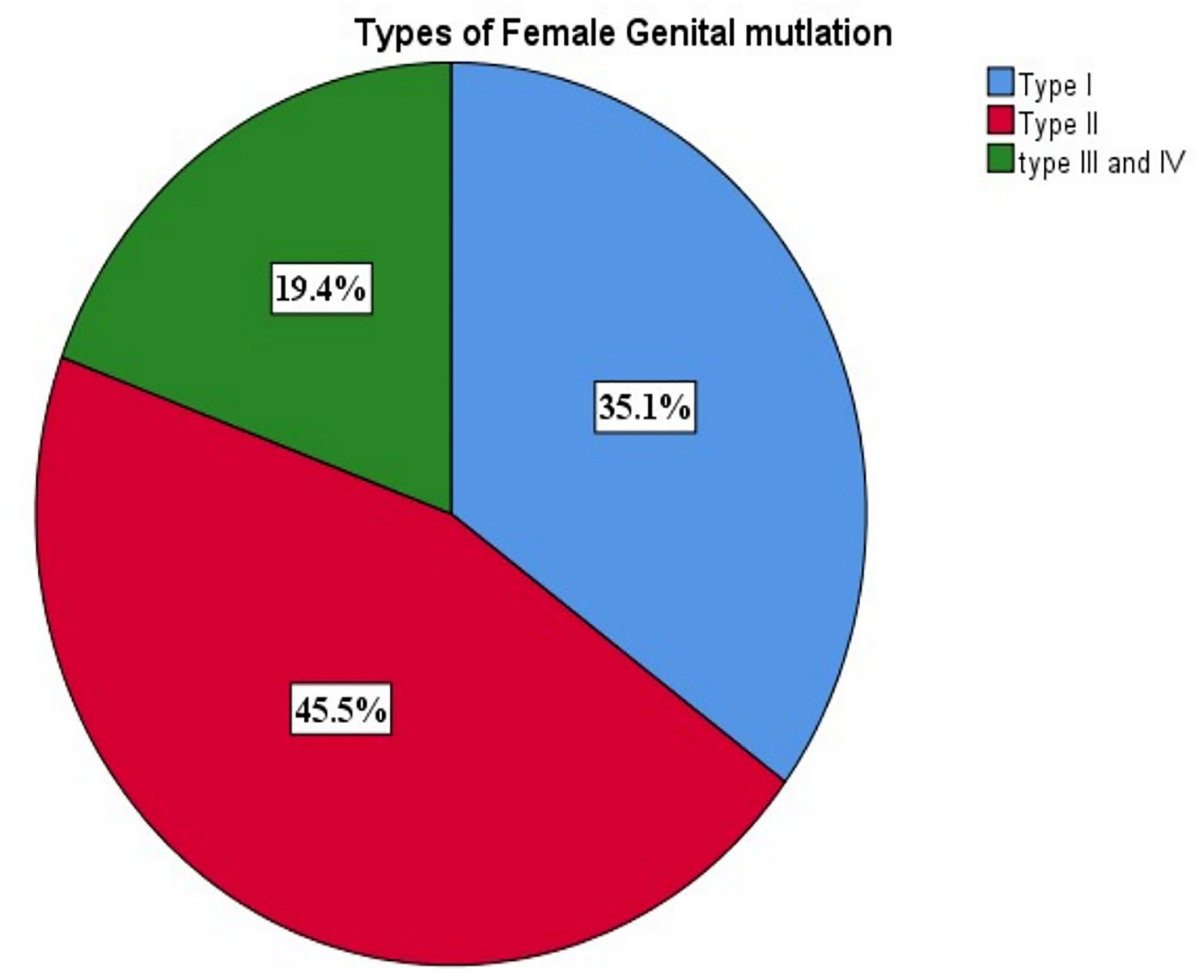
Types of female genital mutilation among women who gave birth vaginally at public health facilities in Jigjiga town, eastern Ethiopia, 2022 (n = 279).

Of these, 98(35.1%) had FGM type I, FGM-II 127(45. 5%), and 54(19.4%) had FGM types III or IV respectively (Fig 2).

Regarding fetal condition, 369(87.4%) of all newborns were born at term (between 37 to 42 completed weeks), and 299 (70.9%) of the newborns had normal birth weight. The majority 378 (89.6%) had a cephalic presentation during labor and delivery, while 44(10.4%) of the fetus had a breech presentation. Non-reassuring fetal heart rate pattern during 2^nd^ stage of labor was documented among (6.4%, n = 27) mothers (Table 3).

**Table 3:** Fetal related characteristics of the respondents at public health facilities in Jigjiga town, Somali regional state, eastern Ethiopia, 2022.

Variables	Categories	n	%
NRFHP on 2^nd^ stage of labor	Yes	27	6.4
	No	395	93.6
Fetal presentation	Cephalic	378	89.6
	Breech	44	10.4
Sex of Newborn	Male	188	44.5
	Female	234	55.5
Gestational age	Preterm	34	8.1
	Term	369	87.4
	Post term	19	4.5
Birth weight	Low birth weight	95	2.5
	Normal birth weight	299	70.9
	Macrosomia	28	6.6

**NB:** NRFHP = Non Reassuring Fetal Heart Rate Pattern.

Concerning mode of delivery, 359(85.1) and 63 (14.9%) of women gave birth through spontaneous vaginal delivery and assisted by instrument (vacuum/forceps) respectively. Likewise, 348 (82.2%) had a spontaneous onset of labor and 74 (17.8%) had induced labor. The majority 344(81.5%) had clear amniotic fluid during the second stage of labor. About 362(86%) of the study participants’ second stage of labor lasted for less than 90 minutes (Table 4).

**Table 4:** Labor and Delivery related characteristics of the respondents at public health facilities in Jigjiga town, Somali regional state, eastern Ethiopia, 2022.

Variables	Categories	Episiotomy	
		Yes (%)	No (%)
**Mode of delivery**			
	SVD	179(49.5%)	183(50.5%)
	Instrumental	43(71.7%)	17(28.3%)
**Delivery time**			
	Day	102(50.0%)	102(50.0%)
	Night	120(55.1%)	98(44.9%)
**Duration of 2nd stage**			
	<90 Minutes	175(48.2%)	188(51.8%)
	≥ 90 minutes	47(79.7%)	12(20.3%)
**Onset of labor**			
	Spontaneous	165(47.4%)	183(52.6%)
	Induced	57(77.0%)	17(23.0%)
**Amniotic Fluid status**			
	Clear	167(48.0%)	177(52.0%)
	Meconium stained	55(73.1%)	23(26.9%)
**Birth attendant’s profession**			
	Midwives	174(50.0%)	174(50.0%)
	Others	48(67.6%)	23(32.4%)
**Health Facility type**			
	Teaching Hospital	97(58.8)	68(41.2%)
	Non-teaching hospital	102(50.5%)	100(49.5%)
	Health Center	23(41.8%)	32(58.2%)

*Other health professionals: medical interns, residents, senior specialists, nurses and public health officers.

### Women’s episiotomy experience

**Fig 3:**
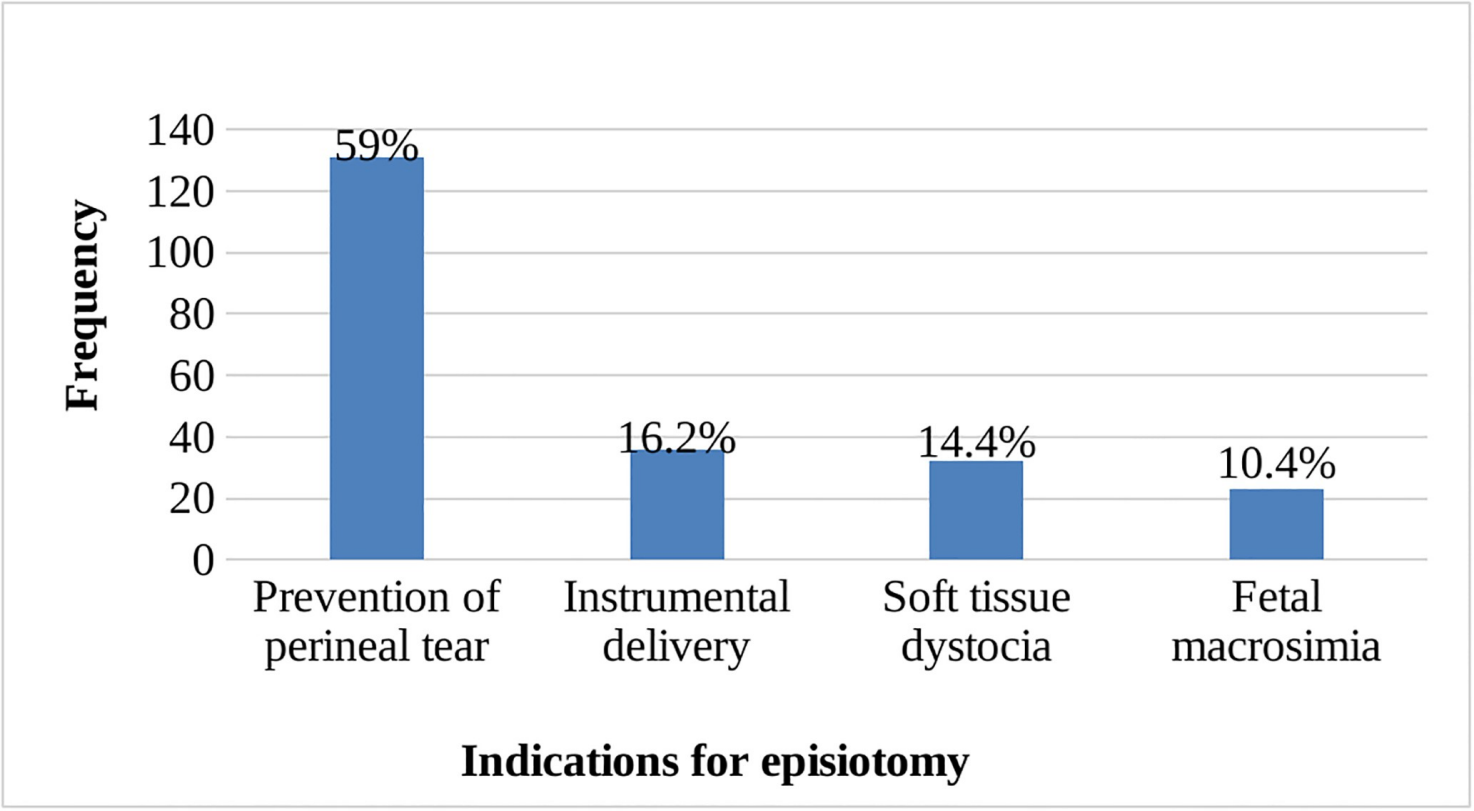
Indications for episiotomy among laboring women at public health facilities of Jigjiga town, eastern Ethiopia, 2022(n = 222).

More than half of the women (52.6%, 95% CI: 47.8%, 57.50%) experienced episiotomy incisions. The majority of the women 218(98.2%) had mediolateral incisions. Preventing perineal tear 131(59%) was the most common indication followed by of use episiotomy to assist instrumental delivery 36(16.2%) as documented by caregivers (Fig 3).

### Factors associated with episiotomy

After adjusting for confounders, a maternal complication in the current pregnancy, FGM, newborn’s birth weight, labor onset, amniotic fluid status, duration of the second stage, and mode of delivery remained significant in the final multivariable analysis at a p-value < 0.05.

This study shows that women who had an obstetric complication during their current pregnancy were 3.92 times more likely to undergo episiotomy during delivery compared to those women who had no complications (AOR:3.92, 95% CI: 1.59, 9.68). Women with macrosomic babies were 4.30 times more likely to have episiotomy experience as compared with women with low birthweight babies (AOR: 4.30, 95% CI: 1.53, 12.04). Furthermore, women with induced labor were 3.1 times more likely to incur episiotomy procedures than those with spontaneous onset of labor (AOR: 3.10, 95% CI: 1.62, 5.93). Women with meconium-stained amniotic fluid during the second stage of labor were almost 2.1 times more likely to have an episiotomy procedure as compared to women with clear amniotic fluid (AOR:2.10, 95% CI: 1.14, 3.88). Women whose second stage of labor lasted 90 minutes or more were 3 times more likely to have an episiotomy during delivery than those whose second stage of labor lasted less than 90 minutes (AOR:3.09, 95% CI: 1.53, 6.23). Similarly, women who gave birth by assisted instrumental delivery (vacuum or forceps) were 2. 69 times more likely to be exposed to episiotomy than those who gave birth through spontaneous vaginal delivery (AOR: 2.69, 95%, CI: 1.39, 5.19). Women who had FGM scar were 2.91 times more likely to be incised during delivery than those women who had no FGM scar (AOR: 2.91, 95% CI: (1.83,4.64) (Table 5).

**Table 5:** Factors associated with episiotomy at public health facilities in Jigjiga town, eastern Ethiopia, May 01 to June 30, 2022 (n = 422).

Variables	Episiotomy		COR (95% CI)	AOR (95% CI)
	Yes (%)	No (%)		
**Obstetric complications**				
Yes	29(80.6%)	7(19.4%)	4.14(2.06,12.1)	**3.92(1.59,9.68)** *
No	193(50%)	193(50%)	1	1
**Female Genital Mutilation**				
Yes	169(60.6%)	110(39.4%)	2.61(1.72.3.95)	**2.91(1.83,4.64)** *
No	53 (37.1%)	90(62.9%)	1	1
**Newborn’s birth weight**				
Low birth weight	36 (37.9%)	59(62.15%)	1	1
Normal birth weight	166(55.5%)	133(44.5%)	2.28(1.52,3.41)	2.04(1.20,3.45)
Macrosomia	20(71.4%)	8(28.6%)	3.59(1.51,8.56)	**4.30(1.53,12.04)** *
**Labor onset**				
Spontaneous	165(47.4%)	183(52.3%)	1	1
Induced	57(77.0%)	17(23.0%)	3.72(2.08,6.65)	**3.10 (1.62,5.93)** *
**Amniotic fluid Status**				
Clear	167(58.8%)	177(41.2%)	1	1
Meconium stained	55(70.5%)	23(29.5%)	2.53(1.54,4.36)	**2.10(1.14,3.88)** *
**Duration of 2^nd^ stage of labor**				
<90 minutes	175(48.2%)	188(51.8%)	1	1
≥90 minutes	47(79.7%)	12(20.3%)	4.21(2.16,8.19)	**3.09(1.53,6.23)** *
**Mode of delivery**				
SVD	179(49.4%)	183(50.6%)	1	1
Instrumental	43(71.7%)	17(28.3%)	2.59(1.42,4.70)	**2.69(1.39,5.19)** *

Significant at:

*P<0.05 and 1 = constant.

## Discussion

The present study determined the prevalence of episiotomy and its associated factors among women who gave birth at public health facilities in Jigjiga town, Somali Regional State, eastern Ethiopia.

The prevalence of episiotomy in this study was found to be 52.6% (95% CI: 47.8%, 57.50%). Obstetric complications during the current pregnancy, FGM scar, birth weight ≥4000gm, induction of labor, meconium-stained amniotic fluid, duration of the second stage of labor ≥90 minutes and instrumental delivery were factors significantly associated with episiotomy in the study area.

The proportion of episiotomy in this study is consistent with the study report of 49.4% in Spain [30], 52.2% in Saudi Arabia [31], 44.0% in China [23], and 47.7% in Ethiopia [24]. But the rate of episiotomy in this study is higher than the recommended value by the WHO (10%) [16]. It was also higher than the previous study findings conducted in the United States (9.4%) [32], Palestine (28.7%) [33], Democratic Republic of Congo (20.4%) [34], other studies conducted in Addis Ababa (35.2%) [19], Bahir Dar (41.1%) [35], and in Eastern Ethiopia [25]. This discrepancy may result from the adoption or implementation of clinical practice guidelines in those countries or regions which promote a restricted use of episiotomies.

Another explanation for the higher prevalence of episiotomy in our study could be a contribution from the high female genital mutilation rates (66.1%) in the study area which increased risk of episiotomy associated with FGM [28].

On the other hand, this study’s finding was lower than the previous study findings conducted in Romania (71.4%) [27], Uganda (73.0%) [36], Ethiopia (68.0%) in Arbaminch [20] and 65.4% in Saint Paul’s Millennium Medical College [29]. This variation might be explained by the difference in study area, study facilities or settings. For example Saint Paul’s hospital is a tertiary level hospital which deals with referral cases of high risk women with obstetric complications.

Concerning factors associated with episiotomies, women who had obstetric complications in their last pregnancy were about 3.9 times more likely to have episiotomies than those without complications during the current pregnancy. This study’s findings were similar to the results of studies conducted in Ethiopia [29,37], and China [38]. This may be due to the practice of episiotomies to shorten the second stage of labor, alleviating the woman and fetus from further complications.

In the present study, FGM was found to be a non-obstetric associated factor that was strongly associated with episiotomy. Women who had FGM were about 2.9 times more likely to have an episiotomy as compared to those women without FGM. This is supported by other study findings, which showed the increased risk of episiotomy associated with FGM [28,39]. This could be explained as the scar tissue formed by FGM narrows the vaginal outlet or orifice, which may necessitate an episiotomy to prevent obstructed labor and extensive perineal lacerations.

This study also found that women with macrosomic babies were 4.3 times more likely to have episiotomies as compared to women with LBW babies. This study’s finding is in line with the study conducted in China [22], France [40] and Ethiopia [20,29] which reported macrosomia as independent risk factor for episiotomy practice. This can be because the larger size of the fetus, the narrower the birth canal, and an episiotomy might be needed to get adequate space due to fear of perineal laceration. Furthermore fetal macrosomia can result in prolonged labor, operative delivery, perineal trauma, shoulder dystocia, birth trauma, and meconium aspiration, which may necessitate an episiotomy, to minimize severe maternal and fetal complications [40,41].This study’s finding implied that clinicians would tend to perform episiotomy for a mother if they assumed the fetal weight was higher.

This study revealed that women who had induced labor were about 3.1 times more likely to have an episiotomy as compared to women with spontaneous labor onset. This finding agrees with the study findings done in Romania [27] and Ethiopia [37,42]. This can be explained by labor induction causing strong uterine contractions that alter the fetal heartbeat pattern, resulting in non-reassuring fetal heartbeat pattern, which may necessitate an episiotomy to shorten the second stage of labor and save the life of the baby [43]. Appropriate use of the induction protocol and close maternal and fetal monitoring are advised during labor and delivery.

In the present study, women who had meconium-stained amniotic fluid were 2.1 times more likely to have an episiotomy as compared to those whose amniotic fluid was clear. This finding is congruent with the study done in France [44]. This is due to the fact that an episiotomy could be done to shorten the second stage of labor and prevent fetal heart rate abnormalities caused by meconium aspiration [45].

Likewise, women whose second stage of labor lasted ≥90 minutes were about 3 times more likely to have an episiotomy as compared to those women whose second stage of labor lasted <90 minutes. This finding is consistent with the findings from other studies done in Uganda [36], Ethiopia [19], and China [22]. This can be justified by the fact that a prolonged second stage of labor can result in maternal exhaustion and increased perineal laceration, which may put the fetus and the women at risk [46]. Maternal resuscitation, avoiding the early artificial rupture of the membrane, and close labor follow-up may reduce the rate of episiotomy.

This study also found a significant association between the mode of delivery and episiotomy. Women who gave birth by vacuum or forceps were nearly 2.7 times more likely to have an episiotomy as compared to those who gave birth spontaneously. This study’s finding is in line with the result of study done in Addis Ababa [29] and Metema, Ethiopia [37]. This study’s finding is also supported by the study, which showed that women who had an episiotomy during instrumental delivery had an 86% lower risk of maternal complications compared to women without an episiotomy [47]. Therefore, the application of instrumental delivery based on the pre-requisite and indications minimizes perineal tears and the need for an episiotomy.

### Strengths and limitations of the study

As strength, this study included all public health facilities in the study area to make the study’s findings representative. Furthermore, some variables that could not be addressed by interview and chart review were addressed by an observational checklist. Despite its representativeness, the cross-sectional nature of the study makes it difficult to determine cause-and-effect relationships. Recall bias might be introduced since some variables such as past obstetric or gynecologic history (history of abortion, age at first pregnancy, and history of operative delivery) were assessed based on maternal self-report.

## Conclusion

Episiotomy practice in the study area is unacceptably higher than the recommended level of episiotomy by WHO. The most common type of episiotomy in this study was mediolateral incision. Obstetric complications during the current pregnancy, instrumental delivery, FGM, birthweight, induction of labor, meconium-stained amniotic fluid, and longer duration of the second stage were identified independent predictors for episiotomy. In addition to the known obstetric indications, female genital mutilation scar increased the risk of women’s experience of episiotomy. Therefore, intervention should be tailored to avoid female genital mutilation and other significantly associated obstetric factors in the study area to reduce women’s experience of episiotomy in the future generation.

## Supporting information

S1 File Episiotomy dataset. (SAV)

S2 File Consent form and data collection tool for episiotomy. (DOCX)
