# Supplementary material for: Prevalence of episiotomy and associated factors among women who gave birth at public health facilities in Jigjiga town, eastern Ethiopia: A cross-sectional study
Source: PLOS Glob Public Health. 2024 May 20;4(5):e0003216. doi: 10.1371/journal.pgph.0003216 (PMC11104620; doi:10.1371/journal.pgph.0003216)
Supplement: S2 File — (DOCX) [file pgph.0003216.s002.docx]

## Consent form

Good morning/afternoon, my name is_________­­___________ I am one of data collectors from Haramaya University, College of Health and Medical Science, and school of Nursing and Midwifery for the study entitled “Episiotomy experience and its associated factors among mothers who gave birth at public health facilities in Jigjiga town, Somali region, eastern Ethiopia.2022” . The goal of this study is to determine episiotomy prevalence and its associated factors among women who give birth at public health facilities in Jigjiga town. The findings of this study can be of a paramount importance for health facility, zonal and regional health offices to plan intervention programs and, to increase community awareness that may improve maternal and neonatal health related to labor and delivery. The information collected from you will be confidential. There will be no information that will identify your personality in particular. You have the right to continue or to withdraw from the study at any time. The findings of the study will be general for the study population and will not reflect anything particular of individual person. The questionnaire will be coded to exclude showing names.

**Declaration of informed voluntary consent:** I have read /read to me the participant information sheet. I have clearly understood the purpose of the research, the procedures, the risks and benefits, issues to confidentiality, the rights of participating and contact address for any queries. I have been given the opportunity to ask questions for things that may be unclear. I was informed that I have the right to stop from the study at any time. Therefore, I declare my voluntary consent to participate in this study with my signature as indicated below.

Participant’s signature _______________Date___________

Data collector signature: ____________ Date___________

## Questionnaires (English Version)

| Date­­­­­­­­­­­­­­­­­­­­­­­­­­­­­­­­­­­­­­­­­­­­­______ ________Health Facility ___________________Generated Code___________________ | | | | | |
| --- | --- | --- | --- | --- | --- |
| S/N | | | Questions | Responses | Skip rule |
| **I: Sociodemographic characteristics** | | | | |  |
|  | How old are you? (Age in Years) | | | ______________ |  |
|  | Where is your Place of residence | | | 1. Urban 2. Rural |  |
|  | What is your current marital status? | | | 1. Single 2. Married 3. Divorced 4. Widowed | If single skip  Skip question 4 |
|  | Age at first Marriage (Years)? | | | _______________ |  |
|  | What is your ethnicity | | | 1. Somali 2. Amhara 3. Oromo 4. Others (specify)_________ |  |
|  | What is your religion you follow? | | | 1. Muslim 2. Orthodox 3. Protestant 4. Others(specify)____ |  |
|  | What is your educational level? | | | 1. No formal education 2. Primary level 3. Secondary level and above |  |
|  | What is your occupation? | | | 1. House wife 2. Government employee 3. Merchant 4. Farmer 5. Others (specify)___________ |  |
| **II Maternal characteristics** | | | | |  |
| 201 | | Do you have history of previous delivery after 28 completed weeks of gestation? | | 1. Yes 2. No | If the answer is “NO” skip to 204 |
| 202 | | If “Yes” how many | | ___________ |  |
| 203 | | For How long you wait for each of the next pregnancy? (in years) | | ________________ |  |
| 204 | | Do you have previous history of previous history of problem during pregnancy? | | 1. Yes 2. No | If “No” skip to  207 |
| 205 | | If “Yes” which problem did you face? | | 1. Vaginal bleeding 2. Anemia 3. Other specify_____ |  |
| 206 | | Do you have previous history of abortion? | | 1. Yes 2. No | If “No” skip to 208 |
| 207 | | If “Yes” how many times? | | 1. _________ |  |
| 208 | | Do you have history Antenatal Care follow-up in the current pregnancy? | | 1. Yes 2. No | If “NO “skip to 210 |
| 1. 204   209 | | If “YES” how many times | | 1. ________ |  |
| 1. 206   210 | | Do you have history of any medical disorder during pregnancy? | | 1. Yes 2. No | If “No” skip 211 |
| 1. 207   211 | | If “Yes” which of the following? | | 1. Maternal hypertension 2. Maternal diabetes 3. Others(specify)______________ |  |

| S/N | Question | Response | Remark |
| --- | --- | --- | --- |
| **II Maternal characteristics** | | | |
| 208 | Female Genital Mutilation | 1. Yes 2. No | If “No” skip to 210 |
| 209 | If “Yes” which Type? | 1. Type I 2. Type II 3. Type III and IV |  |
| 210 | Episiotomy performed? | 1. Yes 2. No | If “No” skip to 301 |
| 211 | If “Yes” which type? | 1. Midline 2. Mediolateral 3. Other (specify_____ |  |
| 212 | What is the indication to perform episiotomy | 1. Prevention of tear 2. instrumental delivery 3. Fetal macrosomia 4. Other (specify)____ |  |
| **III: Fetal Related characteristics** | | |  |
| 301 | Gestational Age for current  Pregnancy (completed weeks) | 1. _____________ |  |
| 302 | Fetal distress during second stage of labor? | 1. Yes 2. No |  |
| 303 | Presenting part of the fetus at birth | 1. Cephalic 2. Breech 3. Other specify_____ |  |
| 304 | Sex of the baby | 1. Male 2. Female |  |
| 305 | Weight of the neonate (in gm) | _________________ |  |
| **V: Labor and delivery related characteristics** | | |  |
| 401 | Onset of labor | 1. Spontaneous 2. Induced |  |
| 402 | Liquor status | 1. Clear 2. Meconium stained |  |
| 403 | Duration of second stage (Minutes) | _________________ |  |
| 404 | Mode of delivery | 1. SVD 2. Instrumental |  |
| 405 | Time of delivery | 1. Day 2. Night |  |
| 406 | Type of birth attendant | 1. Midwives 2. Other health care providers |  |
| 407 | Health Facility Status | 1. Teaching Hospital 2. Non-teaching Hospital 3. Health center |  |

**Thank you for your participation in this study.**
